# Cutaneous larva migrans in Canadian travellers returning from the Caribbean: A 10-year surveillance analysis from CanTravNet

**DOI:** 10.14745/ccdr.v51i05a04

**Published:** 2025-05-01

**Authors:** Andrea Boggild, Rachel Bierbrier, Michael Libman, Cedric Yansouni, Anne McCarthy, Jan Hajek, Wayne Ghesquiere, Yazdan Mirzanejad, Katherine Plewes, Jean Vincelette, Susan Kuhn, Pierre Plourde, Christina Greenaway, Kevin Kain, Shaun Morris, Sapha Barkati

**Affiliations:** 1 Division of Infectious Diseases, Department of Medicine, University Health Network and the University of Toronto, Toronto, ON; 2 Institute of Medical Science, University of Toronto, Toronto, ON; 3 Division of Dermatology, McGill University, Montréal, QC; 4 J. D. MacLean Centre for Tropical & Geographic Medicine, McGill University, Montréal, QC; 5 Division of Infectious Diseases, Ottawa Hospital and the University of Ottawa, Ottawa, ON; 6 Division of Infectious Diseases, University of British Columbia, Vancouver, BC; 7 Infectious Diseases, Vancouver Island Health Authority, Department of Medicine, University of British Columbia, Victoria, BC; 8 Fraser Health, Surrey, BC; 9 Centre hospitalier de l’Université de Montréal, Université de Montréal, Montréal, QC; 10 Section of Pediatric Infectious Diseases, Department of Pediatrics, Alberta Children’s Hospital and the University of Calgary, Calgary, AB; 11 Travel Health & Tropical Medicine Services, Population and Public Health Program, Winnipeg Regional Health Authority, Winnipeg, MB; 12 Division of Infectious Diseases, Jewish General Hospital, McGill University, Montréal, QC; 13 SAR Laboratories, Sandra Rotman Centre for Global Health, Toronto, ON; 14 Department of Pediatrics, Faculty of Medicine, University of Toronto, Toronto, ON; 15 Division of Infectious Diseases, Hospital for Sick Children, Toronto, ON

**Keywords:** ivermectin, dermatosis, zoonosis, helminthic infection, tropical, GeoSentinel, tourism

## Abstract

**Background:**

Cutaneous larva migrans (CLM) is one of the most common dermatoses affecting travellers to the tropics.

**Objective:**

To describe demographic and travel correlates of travellers returning to Canada from the Caribbean with CLM over a 10-year pre-pandemic period.

**Methods:**

Demographic and travel-related data on ill travellers encountered either during or after completion of their travel/migration and seen in any of eight CanTravNet sites from January 1, 2009, to December 31, 2018, with a final diagnosis of CLM were extracted and analyzed. During this time, access to first-line therapy, ivermectin, was available via Health Canada’s Special Access Programme.

**Results:**

Of 17,644 travellers presenting to CanTravNet over the enrolment period, 328 (1.9%) returned from the Caribbean with CLM. The median age of travellers with CLM was 34 years (interquartile range: 25–50 years), with females accounting for 58% of cases. Ninety-five percent (n=313) travelled for tourism. Jamaica was the most common source country, with 216 cases (67%), followed by Barbados (n=27, 8%) and the Dominican Republic (n=23, 7%). Cases in 2018 were imported predominantly from Jamaica (n=58, 73%) and the Dominican Republic (n=12, 15%). Age, sex and purpose of travel were similar across years. The percentage of all imported cases of CLM that originated from the Caribbean increased from 9% in 2016 to 24.5% in 2018.

**Conclusion:**

Proportions and absolute numbers of CLM in travellers returning to Canada from the Caribbean are increasing. Improved awareness of this common dermatosis among physicians and travellers, as well as improved access to effective therapies, will reduce associated morbidity.

## Introduction

Cutaneous larva migrans (CLM) is a zoonotic helminthic infection caused most often by *Ancylostoma caninum, Ancylostoma braziliense* and *Uncinaria stenocephala* ((1)). Animal hookworms live in the intestines of dogs and cats, and their eggs are released in the feces, hatching within a day in soil or sand. Within a week, the eggs develop into larvae capable of infecting humans through direct skin contact. Cutaneous larva migrans is acquired via transcutaneous penetration of the larval helminths into intact skin. Humans are accidental hosts and organisms responsible for CLM lack digestive enzymes enabling penetration of human basement membrane ((1)). Humans cannot transmit the infection to other humans or animals. Although not endemic to Canada, the infection is imported by travellers who typically consult primary care, emergency departments, dermatology, infectious disease and tropical medicine experts for management.

Cutaneous larva migrans is a clinical diagnosis characterized by the classic presentation of a pruritic serpiginous tract that advances on average of 2–3 mm per day ((2)). The typical distribution involves areas exposed to contaminated soil or sand, usually the foot or buttocks, but can be extensive ((3,4)). Patients often report intense pruritus that is disruptive to sleep, concentration and quality of life, and can persist for the duration of the helminth lifespan, on average five to six weeks, but upwards of a year or more in some patients ((5,6)).

The GeoSentinel Surveillance Network is a multinational provider-based surveillance system of international travellers and migrants presenting to travel/tropical medicine clinics ((7)). There are eight Canadian sites of participation in GeoSentinel spread across major urban centers within the country. The Canadian sites constitute CanTravNet, a national surveillance network with a catchment area of approximately 45% of the Canadian population. The Network serves as a resource to identify emerging pathogens, monitor disease activity and analyze population-based data.

Cutaneous larva migrans is the most commonly reported cutaneous disorder in returned travellers captured by the GeoSentinel Network ((8)). Notably, CLM can be acquired through travel to almost every continent, including sub-tropical areas of North America ((9,10)). From 2009 through 2011, 7% of travel-related diagnoses in Canada were for CLM in travellers returning from the Caribbean ((11)). Since the last study, no additional research has described the epidemiology of CLM in Canadian travellers returning from abroad.

The objective of this surveillance report is to describe demographic and travel correlates of travellers returning to Canada from the Caribbean with CLM over a 10-year pre-pandemic period during which time access to the first-line treatment in Canada, ivermectin, was uniformly available via the Special Access Programme of Health Canada. Better characterization of this disease in Canadian travellers may prompt expansion of resources available through Health Canada to treat this common travel-related dermatosis and increase awareness of its diagnosis among healthcare providers.

## Methods

### Data source

Eight Canadian sites in large urban centres from five provinces (British Columbia, Alberta, Manitoba, Ontario and Québec), also belonging to the GeoSentinel Global Surveillance Network, constitute CanTravNet ((11)). CanTravNet sites are staffed by licensed infectious disease physicians with expertise in travel-acquired illnesses and tropical medicine. Demographic and travel-related data were collected using the data platform of the GeoSentinel Surveillance Network (for additional details, see https://geosentinel.org) ((7,12,13)). The diagnosis of CLM is made clinically and the site director can choose this diagnosis from the 475 options classified as either etiologic or syndromic. The GeoSentinel data collection protocol is reviewed by the institutional review board officer at the National Center for Emerging and Zoonotic Infectious Diseases at the US Centers for Disease Control and Prevention and has been classified as public health surveillance, not human subject research requiring approval from institutional review boards. Local site institutional review board approval was obtained where required. Final diagnoses include specific etiologies (e.g., CLM) and syndromes (e.g., rash).

### Definitions and classifications

Seven travel purpose designations were used, including tourism, business, missionary/volunteer research/aid work, visiting friends and relatives, education and planned medical care; or “migrants”, which captured those travelling for immigration, refugee settlement or asylum-seeking. Visiting friends and relatives travel is as defined by Leder *et al.*, though classification of the purpose of travel is based on the clinician’s best judgment when there was more than a single possible location of exposure ((12)).

### Inclusion criteria

Demographic, clinical and travel-related data on ill Canadians and migrants encountered either during or after completion of their travel/migration and seen in any of the eight CanTravNet sites from January 1, 2009, to December 31, 2018, with a final diagnosis of CLM were extracted and analyzed. The decade for inclusion was selected as a representative pre-pandemic decade, during which time access to the drug of choice, ivermectin, was uniformly available via the Special Access Programme of Health Canada.

### Analysis

Extracted data were managed in a Microsoft Access database and travellers were described by purpose of travel, demographics and itinerary. Data on sex and travel region were available for all travellers, and all were included in the analyses even if travellers’ age or their specific country of travel were missing. Descriptive analyses including medians with interquartile ranges [IQR] and proportions were calculated for continuous and categorical variables, respectively. The significance of the trend in case distribution over the year was assessed using a Poisson regression model. All statistical computations were performed using Stata/BE 17.0 (StataCorp, College Station, Texas, United States).

## Results

**Figure 1:**
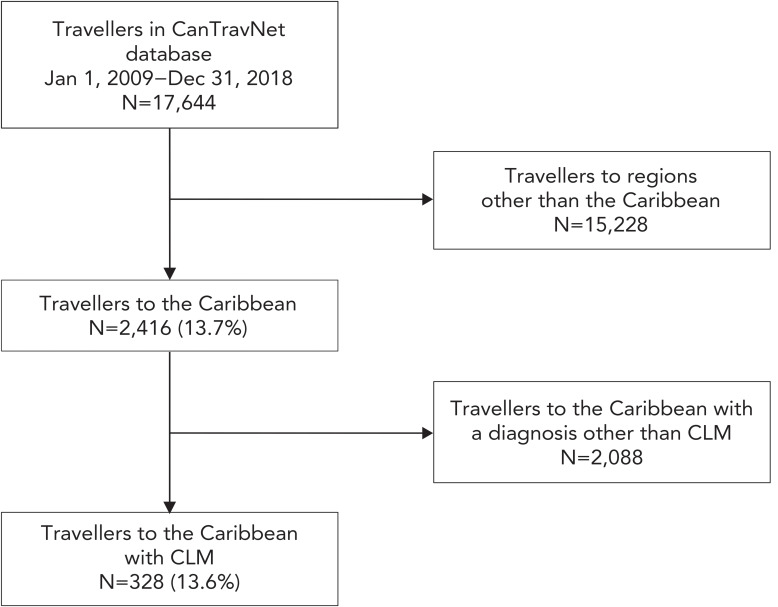
Flow diagram of cutaneous larva migrans in Canadian travellers returning from the Caribbean, 2009–2018 Abbreviation: CLM, cutaneous larva migrans

A total of 17,644 travellers presented to a CanTravNet site between January 1, 2009, and December 31, 2018. Of all recorded travellers, 2,416 (13.7%) returned from travel to the Caribbean. Of travellers to the Caribbean seeking post-travel medical care, 328/2,416 (13.6%) were diagnosed with CLM (**Figure 1**) Median age of the returned travellers with CLM was 34 years (IQR: 25–50 years), with males accounting for 42% (n=139) and females for 58% (n=189) of cases (**Table 1**). Cases were more common in the 18–34 years and 35–65 years age groups, which together accounted for 80% of the total cases. While cases were reported throughout the year, there were consistently a higher number of cases between December and March, coinciding with the Canadian winter season.

**Table 1:** Cases of cutaneous larva migrans among Canadian travellers returning from the Caribbean by year

Year of import	Number of cases (%)^a^	Age, years, median (IQR)	Sex, M/F N (%)	Travelling for tourism, N (%)	Top three source countries (N; %)		
					First place	Second place	Third place^b^
2018	81 (24.5%)	32.0 (19–50)	39/42 (48%/52%)	80 (99%)	Jamaica (58; 73%)	Dominican Republic (12; 15%)	Antigua and Barbuda (2; 3%) Barbados (2; 3%) Saint Lucia (2; 3%)
2017	63 (25.7%)	39.0 (27–53)	24/39 (38%/62%)	58 (92%)	Jamaica (45; 71%)	Dominican Republic (8; 13%)	Barbados (3; 5%) Martinique (3; 5%)
2016	27 (9.0%)	33.0 (12–53)	7/20 (26%/74%)	26 (96%)	Jamaica (15; 58%)	Cuba (3; 12%)	Saint Lucia (2; 8%) Saint-Martin (2; 8%)
2015	23 (9.2%)	38.0 (27–52)	9/14 (39%/61%)	23 (100%)	Jamaica (15; 65%)	Barbados (2; 9%) Cuba (2; 9%) Grenada (2; 9%)	Antigua and Barbuda (1; 8%) Saint Vincent and the Grenadines (1; 8%)
2014	32 (11.9%)	38.0 (20–50)	13/19 (41%/59%)	31 (97%)	Jamaica (20; 65%)	Cuba (7; 23%)	Barbados (1; 3%) Bahamas (1; 3%) Martinique (1; 3%) Saint-Martin (1; 3%)
2013	36 (13.4%)	37.5 (25–49)	17/19 (47%/53%)	32 (89%)	Jamaica (23; 64%)	Barbados (6; 17%)	Saint Lucia (5; 14%)
2012	23 (11.0%)	32.0 (25–44)	10/13 (43%/57%)	23 (100%)	Jamaica (20; 87%)	Barbados (1; 4%) Cuba (1; 4%) Guadeloupe (1; 4%)	-
2011	17 (9.1%)	37.0 (29–48)	8/9 (47%/53%)	15 (88%)	Barbados (7; 41%)	Jamaica (6; 35%)	Cuba (4; 18%)
2010	11 (6.5%)	36.0 (26–50)	6/5 (55%/45%)	11 (100%)	Jamaica (8; 72%)	Saint Lucia (2; 18%)	Aruba (1; 9%)
2009	15 (7.9%)	28.0 (15–36)	6/9 (40%/60%)	14 (93%)	Jamaica (6; 40%)	Barbados (4; 27%)	Cuba (2; 13%) Guadeloupe (2; 13%)
Total^c^	328 (13.6%)	34.0 (25–50)	139/189 (42%/58%)	313 (95%)	Jamaica (216; 67%)	Barbados (27; 8%)	Dominican Republic (23; 7%)

Abbreviations: F, female; IQR, interquartile range; M, male; -, not applicable

^a^ Percentage derived from the denominator of total travellers to the Caribbean each year presenting to a CanTravNet site

^b^ All countries noted were tied for third place

^c^ Total cases of cutaneous larva migrans seen at CanTravNet sites between January 1, 2009, and December 31, 2018=570; thus, cases from the Caribbean represent 57.5% of total cases

The purpose of travel was tourism in 95% (n=313) of the travellers with CLM. Jamaica was the most well-represented source country, accounting for 216 cases (66.7%), followed by Barbados (n=27, 8.3%) and the Dominican Republic (n=23, 7.1%) (**Table 2**).

**Table 2:** Source Caribbean countries for cases of cutaneous larva migrans evaluated at CanTravNet sites, January 2009–December 2018

Country	Number	Percentage
Jamaica	216	66.7
Barbados	27	8.3
Dominican Republic	23	7.1
Cuba	21	6.5
Saint Lucia	11	3.4
Guadeloupe	4	1.2
Martinique	4	1.2
Saint-Martin	3	0.9
Antigua and Barbuda	3	0.9
Bahamas	2	0.6
Grenada	2	0.6
Turks and Caicos	2	0.6
Cayman Islands	1	0.3
Aruba	1	0.3
Haiti	1	0.3
Puerto Rico	1	0.3
Saint Vincent and the Grenadines	1	0.3
Trinidad and Tobago	1	0.3

The majority of reported CLM cases presented to CanTravNet sites were from Toronto (n=191, 58.2%), followed by Montréal (n=92, 28.1%), Ottawa (n=22, 6.7%) and Calgary (n=13, 4.0%) (**Table 3**).

**Table 3:** Site distribution for cases of cutaneous larva migrans evaluated at CanTravNet sites, January 2009–December 2018

Site	Number	Percentage
Toronto	191	58.2
Montréal	92	28.1
Ottawa	22	6.7
Calgary	13	4.0
Vancouver	9	2.7
Winnipeg	1	0.3

**Figure 2:**
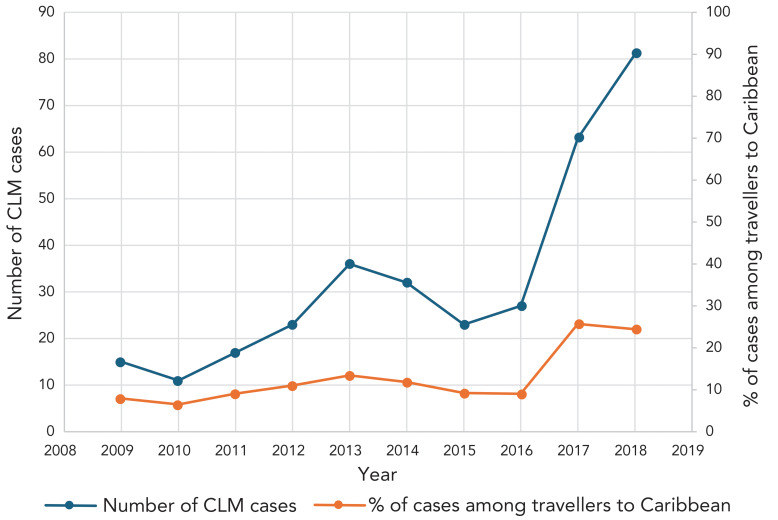
Number and percentage of cases of cutaneous larva migrans among travellers returning from the Caribbean, 2009–2018 Abbreviation: CLM, cutaneous larva migrans

The average number of CLM cases among travellers to the Caribbean increased to 81 in 2018, three times what was reported in 2016 (Table 1). Compared to 2009, there was a fivefold increase in CLM cases in 2018 (Poisson regression coefficient 0.197; 95% CI: 0.156–0.239) (**Figure 2**). Before 2017, the annual proportion of ill returned travellers presenting to a CanTravNet site with CLM from the Caribbean was approximately 6.5%–13.4% (Table 1). In the years 2017 and 2018, however, that proportion increased to 25.7% and 24.5%, respectively (Table 1).

## Discussion

Our surveillance report describes the landscape of CLM in ill Canadian travellers returning from the Caribbean over the course of a decade. Jamaica, Barbados and the Dominican Republic were the most common Caribbean source countries for this helminthic infection. Overall, our data demonstrate an increase in the number of yearly CLM cases, peaking from 2016 through 2018, where recorded CLM cases increased threefold. As noted by Lederman *et al.* and confirmed in our analysis, CLM was most prevalent in the 18–65 years age group; however, our analysis revealed a higher proportion of females with CLM compared to their study. Additionally, both studies identified Barbados and Jamaica as the leading countries ((8)).

Cutaneous disorders (e.g., CLM, arthropod bite reactions, abscesses and allergic reactions) in returning travellers are frequent causes for medical consultation, accounting for up to 18% of visits to specialized travel clinics ((8)). Specifically, CLM accounted for 9.8% of dermatoses in returned travellers who visited the GeoSentinel Surveillance Network clinics from January 1997–February 2006 ((8)). At 25%, it was the most common dermatosis reported in a French cohort of returning travellers from 1991 to 1993 ((14)) and represented 13% of 1,076 dermatological diagnoses in a Canadian cohort from September 2009–September 2012 ((15)). In a previous GeoSentinel study (June 1996–August 2004), CLM was most commonly associated with travel to the Caribbean ((16)). Given that the International Organization of Tourism reported an almost twofold increase in international travel to the Caribbean from 1995 to 2017, clinicians providing care to ill returned travellers may expect to encounter an increasing number of cases of CLM if this travel trend continues ((17)).

Cutaneous larva migrans remains an important travel-related dermatosis. Although usually self-limited, the disease is associated with significant and prolonged morbidity, often over months, related to pruritus, sleep disturbance and secondary bacterial infection, prompting consultation in specialized travel medicine clinics. As reported in prior cohorts of CLM cases in Canada, median duration of symptoms is typically over a month, and can range from less than a week to close to a year before care-seeking and/or effective treatment occurs. Prior to presenting to specialized clinics, many patients receive ineffective (e.g., mebendazole, topical antimicrobials) and potentially harmful (e.g., corticosteroids, oral antimicrobials) therapeutic interventions by other providers ((18,19)).

Treatment of CLM is indicated for those who are symptomatic. Albendazole and ivermectin are the two most recognized treatments for CLM. Topical thiabendazole may also be used ((19)). Although readily available in many regions internationally, access to treatment for CLM in Canada is more limited. In Canada, CLM is considered of little public health significance given the lack of propensity to propagate outside of the accidental host; however, significant morbidity may be associated with those affected by the disease, warranting prompt and effective treatment. Prior to late 2018, physicians were required to apply to Health Canada’s Special Access Programme in order to obtain access to ivermectin, which remains the process for access to albendazole. The application process through the Special Access Programme did not guarantee access to ivermectin and led to delays in treatment initiation ((20,21)). With recent Health Canada approval of ivermectin for strongyloidiasis, off-label access in Canada for CLM has become easier ((22,23)).

The approval of ivermectin by Health Canada was a significant step in improving access to effective medications for travel acquired diseases. Although Canada lacks access to many drugs on the World Health Organization’s essential medication list, specifically those related to management of imported infectious diseases, the approval of ivermectin has enabled more timely access to effective therapy for a variety of helminthiases ((24)). Lack of access to effective therapies for imported infectious diseases is a significant issue given that there were approximately 12 million Canadians returning from international travel and 21.1 million international tourists to Canada in 2018 ((25,26)). Further, Canada is home to many refugees and migrants, who originate from countries endemic to many tropical and travel-related diseases ((27)). Therefore, ongoing advocacy to obtain essential medications is critical to provide first-line patient care in the context of evolving trends in travel and migration among the Canadian population.

Counselling on preventive strategies in travellers is a fundamental component of a pre-departure visit. All physicians offering pre-travel consultations should reiterate the importance of closed-toed shoes and sitting on towels or blankets when contacting sand or soil in areas endemic for CLM. Additionally, those travelling home to visit friends and relatives should be cautioned about the risk of acquiring CLM of the hands and knees via gardening, an activity that also portends risk of strongyloidiasis acquisition. In an outbreak of CLM amongst Canadian travellers in 2000, use of sandals was associated with lower risk of acquiring infection ((28)). Avoidance of excoriation of pruritic areas can reduce the risk of secondary bacterial infection ((29)). For early management to decrease disease morbidity, travellers should be encouraged to visit a medical provider should any skin lesions appear upon return from travel ((29)).

### Limitations

There are several limitations to our study. First, the report is a surveillance analysis of prospectively entered data that are analyzed in a retrospective manner; as such, we are limited to analyzing the data fields present in the surveillance instrument and are unable to obtain more granular exposure details, such as high-risk activities or contact with specific types of animals. The use of surveillance data limits our ability to report full details on clinical presentation, evolution, disease morbidity, treatment selection and response to therapy, as this is not recorded by GeoSentinel’s surveillance instrument. As GeoSentinel only captures data from travel health clinics, our review does not represent all cases of CLM across Canada within our defined period, nor may our findings extend to travellers returning to other home countries. Furthermore, individuals who present to travel health clinics may differ behaviourally, demographically, or socioeconomically from the general population of travellers, thereby potentially introducing selection bias. Additionally, since the drug of choice for treatment of CLM, ivermectin, was approved by Health Canada in late 2018, which coincides with our end date of enrolment, it is uncertain how formulary access to ivermectin by non-specialists may have influenced representation of CLM as a diagnosis in the CanTravNet database. Moreover, given that the database does not contain information on all returning travellers, only those who presented to post-travel clinics for illness, no denominator exists for statistical calculation of incidence rates or absolute risk. Finally, the data presented herein captured a representative decade of cases presenting for care at our centres pre-pandemic and, as such, our ability to comment on the impact of pandemic-related travel cessation and then subsequent partial recovery on the epidemiology of CLM in Canada is limited. Despite limitations, GeoSentinel and CanTravNet remain important surveillance systems that provide valuable data on the epidemiology of travel- and migration-associated infectious diseases.

## Conclusion

Cutaneous larva migrans is a common travel-related dermatosis in Canadians returning from the Caribbean. Cutaneous larva migrans was predominantly observed in the 18–65 years age group, with a greater proportion of females compared to males. Barbados and Jamaica were identified as the most affected countries. With rising frequency of CLM in our clinics, increased awareness among healthcare providers and appropriate counselling of travellers, combined with improved access to effective therapy, can reduce disease morbidity. Overall, CLM is a common, typically self-limited travel-related dermatosis that can cause significant morbidity related to sleep and functional disturbance. Cutaneous larva migrans may continue to emerge in travellers once air travel has recovered to pre-pandemic levels. Moreover, CLM is a zoonotic disease for which large-scale surveillance data on source animals in high-risk areas are lacking. Results from our analysis suggest that CLM may be increasing in frequency in Canadian travellers returning from the Caribbean. Awareness of this common dermatosis in the returning traveller is important for Canadian physicians, so that timely and appropriate therapy can be initiated.
